# Effect of implementing the TAGEET communication model on communication competency among pediatric oncology nurses: a quasi-experimental study

**DOI:** 10.1186/s12912-026-04677-x

**Published:** 2026-04-21

**Authors:** Fadi Zaben, Ayman Hamdan-Mansour

**Affiliations:** 1 Department of Pediatric Nursing, Faculty of Nursing, An-Najah National University, West Bank, Palestine; 2 School of Nursing, The University of Jordan, Amman, Jordan

**Keywords:** Pediatric oncology nursing, Therapeutic communication, Communication skills training, TAGEET communication model, Nursing competency, Family-centered care, Quasi-experimental study

## Abstract

**Background:**

Effective therapeutic communication is critical in pediatric oncology, where nurses must provide accurate and compassionate information to children with cancer and their families. However, communication remains complex and is often hindered by clinical, organizational, and training-related barriers. Structured communication models such as TAGEET (Tune-in, Approach and introduce, Ground self, Engage and respond, End encounter, Tune-out) may enhance nurses’ competencies and strengthen family-centered care.

**Objective:**

To evaluate the effect of implementing the TAGEET communication model on pediatric oncology nurses’ communication competency in practicing effective therapeutic communication with children diagnosed with cancer and their parents.

**Methods:**

A quasi-experimental pretest–posttest interrupted time-series design was conducted in two pediatric oncology hospitals in the West Bank, Palestine. All eligible pediatric oncology nurses (*n* = 39) were recruited using a census approach. Participants received a structured communication skills training program based on the six-step TAGEET communication model, delivered over three weeks (three sessions; total 6 h). Data were collected at baseline (T0), immediately post-intervention (T1), and two months post-intervention (T2). Communication competency was assessed using the validated Arabic version of the Communication Assessment Tool (CAT-15). Repeated measures ANOVA was used to examine changes in communication scores across time points, with statistical significance set at *p* < .05.

**Results:**

Thirty-nine pediatric oncology nurses participated (100% response rate). Baseline communication competency was moderate (3.84 ± 0.46) and improved significantly immediately after the intervention (4.53 ± 0.31) and at two-month follow-up (4.62 ± 0.26). Repeated measures ANOVA showed a significant time effect (*p* < .001, partial η² = 0.782). Pairwise comparisons confirmed significant improvements across all time points (*p* < .001), with large effect sizes (d = 1.78–2.03), indicating sustained gains following the TAGEET intervention.

**Conclusion:**

The TAGEET communication model was associated with improved communication competency among pediatric oncology nurses, particularly in areas related to information exchange, engagement, and shared decision-making. While these findings support the potential value of structured communication training, further research incorporating patient- and parent-reported outcomes and objective measures is needed to determine its impact on patient experience and quality of care.

## Introduction

 Healthcare providers (HCPs), particularly nurses, have a professional and ethical responsibility to provide accurate and compassionate information to pediatric oncology patients and their families [1]. Therefore, using effective therapeutic communication is essential to facilitates patient’s understanding, coping, and treatment adherence [2]. Communication in pediatric oncology is uniquely complex task due to the perception of life-threatening nature of cancer, developmental variations among children, and the need to simultaneously address family emotional needs and decision-making roles [3]. HCPs need to balance delivering sensitive medical information while maintaining hope, supporting parental decision-making, and coordinating multidisciplinary care. This would emphasize the critical importance of developing strong communication skills to effectively convey information and care plans to pediatric oncology patients and their families, fostering clarity, trust, and shared understanding.

Despite its importance, multiple barriers hinder effective communication in pediatric oncology settings. Nurse-related barriers include high workload, limited time, inadequate communication training, staff fatigue, and insufficient institutional support [2, 4]. While, family-related barriers may include health illiteracy, language difficulties, and lack of privacy [5], and organizational and interdisciplinary barriers include unclear professional roles, time pressures, and absence of standardized communication frameworks [6]. These barriers, collectively, can further hinder effective communication processes between patient-families-HCPs leading to poor healthcare outcomes and implicit conflict. Acknowledging the negative impact of poor communication is a significant factor that calls for HCPs to acquire effective communications skills.

Effective communication is a fundamental component of patient-centered care and high-quality healthcare delivery, particularly in pediatric oncology, where nurses often serve as the primary communication link between patients, families, and the multidisciplinary healthcare team [3, 7]. Evidence suggests that clear, empathetic, and structured communication improves family understanding of medical information, enhances treatment adherence, and increases patient and family satisfaction [8]. However, despite its recognized importance, communication practices in pediatric oncology often remain intuitive and experience-based rather than systematically guided [6]. In many clinical settings, including the current study context, communication training is primarily limited to general content delivered during undergraduate nursing education or occasional continuing education activities. Such training is often not standardized nor specifically tailored to the complex, emotionally demanding communication needs of pediatric oncology care. This gap underscores the need for structured, context-specific communication models that can strengthen nurses’ competencies and support more effective therapeutic interactions with pediatric oncology patients and their families.

The TAGEET (Tune-in, Approach and introduce, Ground self, Engage and respond, End encounter, Tune-out) communication model offers a structured and practical approach to addressing these needs. A six-step therapeutic communication strategy-Tune-in, Approach and introduce, Ground self, Engage and respond, End encounter, and Tune-out- it integrate emotional awareness with deliberate stepwise engagement [9]. By guiding nurses through preparation, interaction, and reflection phases, TAGEET reduces uncertainty during emotionally charged encounters and supports purposeful rather than reactive communication [9]. Its systematic structure facilitates both relational (empathy, respect, emotional attunement) and cognitive (clarity of information, checking understanding, collaborative planning) components of communication, making it particularly relevant for pediatric oncology settings where both dimensions are essential. For children and families navigating complex diagnoses and treatment decisions, such a model may enhance trust, encourage participation, and promote shared decision-making, ultimately strengthening family centered care (FCC).

Although previous research has examined communication challenges in healthcare settings, limited evidence exists regarding structured communication interventions in pediatric oncology nursing practice, particularly in Palestine. Few studies have examined whether implementing a structured, culturally adaptable communication model can measurably improve nurses’ communication competency and, in turn, positively influence interactions with children and their families. Given the vulnerability of pediatric oncology patients and the central role nurses play in daily communication, evaluating structured models such as TAGEET is both clinically and ethically imperative. Therefore, this study aims to assess the effect of implementing the TAGEET communication model on nurses’ communication competency in practicing effective communication skills with pediatric oncology patients and their parents.

## Materials and methods

### Study design

A quasi-experimental, pretest–posttest, interrupted one-group time series design was employed. Data were collected at three time points: baseline (T0), immediately post-intervention within 24 h (T1), and two months post-intervention (T2). Data collection was conducted between April and August 2025.

### Setting

The study was conducted in pediatric oncology units within two non-profit specialist hospitals in the West Bank, Palestine. Both hospitals are accredited by the Joint Commission International (JCI), provide comparable oncology services, and operate dedicated pediatric oncology units, ensuring consistency in care context.

Pediatric oncology nurses in these settings are primarily involved in inpatient care, including treatment administration, symptom management, and continuous communication with children and their families across the illness trajectory. Their role includes providing emotional support, clarifying medical information, and reinforcing care plans following physician-led discussions. While nurses may be present during complex conversations such as diagnosis disclosure or prognosis discussions, these are typically led by physicians. Nurses play a critical supportive role during palliative and end-of-life care within the hospital setting. However, structured home visits are not part of routine nursing responsibilities in these settings.

### Sample

A census sampling approach was used to recruit all eligible pediatric oncology nurses working in the selected hospitals. Inclusion criteria required nurses to be currently employed in pediatric oncology units. Nurses with less than six months of experience were excluded to ensure adequate exposure to oncology care processes, communication demands, and interactions with patients and families across different stages of care, including acute treatment and, where applicable, palliative care.

### Intervention

The intervention consisted of a structured communication skills training program based on the TAGEET model, a six-step therapeutic communication framework (Tune-in, Approach and introduce, Ground self, Engage and respond, End encounter, and Tune-out). Prior to this intervention, communication skills training within the participating institutions was largely limited to general education and non-standardized continuing education activities, with minimal focus on the specific communication challenges encountered in pediatric oncology. Therefore, the TAGEET model was introduced as a structured, context-specific intervention designed to enhance nurses’ competency in managing complex and emotionally laden interactions with children and their families. The program aimed to improve nurses’ communication competency through a standardized, evidence-based approach.

Training was delivered by the principal researcher using standardized materials, including lectures, role-play scenarios, and guided discussions. The program was implemented over three weeks in three sessions (2 h each; total = 6 h), conducted in small groups to promote engagement and skill development.

To reinforce learning, participants received a summary handout after training and a follow-up instructional video one month later via the Continuing Education Departments. These strategies supported knowledge retention and sustained application in practice.

### Measurement

Data were collected using standardized self-report questionnaires administered in Arabic. The instrument is translated and culturally adapted in accordance with the World Health Organization Disability Assessment Schedule (WHODAS 2.0) translation guidelines (Version 1.0). A professional English language editor reviewed the original English versions prior to translation to ensure conceptual and linguistic accuracy. The translated instrument is evaluated for clarity, cultural relevance, and contextual appropriateness before administration to participants.

### Communication assessment tool (primary outcome measure)

The Communication Assessment Tool (CAT) was used to evaluate therapeutic communication skills [10]. In this study, the validated Arabic version of the tool was employed [11]. The tool consisted of 15 items rated on a 5-point Likert scale: 1 = poor, 2 = fair, 3 = good, 4 = very good, and 5 = excellent, with total scores ranging from 15 to 75. To adapt the CAT for nursing contexts, minor modifications were made to the instructions and item stems. Specifically, references to “your doctor” or “the doctor” were replaced with “your nurse,” and item 15, which originally referred to “the doctor’s staff,” was revised to “nurses’ staff. In addition, the wording of the scale was refined to reflect nurses’ communication behaviors and competencies [11]. In the context of this quasi-experimental study, the CAT served as the primary outcome measure to evaluate changes in nurses’ communication competency following exposure to the TAGEET model. The CAT has demonstrated strong internal consistency in previous studies (Cronbach’s alpha = 0.84). Internal reliability was reassessed within the present sample to ensure measurement consistency across time points.

### Demographic and professional characteristics

A structured questionnaire collected data on participants’ age, gender, education, marital status, years of experience (general and pediatric oncology), primary position, satisfaction, prior training, and perceived impact of communication on care.

### Data collection procedure

All eligible nurses (*N* = 39) were approached, and all agreed to participate (100% response rate). Participants completed the CAT at baseline (T0), immediately after the intervention (T1), and at two-month follow-up (T2). All participants completed the study at each time point.

### Ethical consideration

Ethical approval was obtained from An-Najah National University (Approval No. Nur.1234, February 15, 2025) and the participating hospitals. The study adhered to the Declaration of Helsinki. Written informed consent was obtained from all participants. Confidentiality was ensured through secure data storage and restricted access. Participation was voluntary, with the right to withdraw at any time without consequences.

### Data analysis

Statistical Package for the Social Sciences (SPSS) 25 version was used to analyze the data. Categorical variables were summarized using frequencies and percentages, while continuous variables were described using measures of central tendency (mean and median) and measures of dispersion (standard deviation, minimum, and maximum). Repeated Measures ANOVA (RM-ANOVA) was used to examine differences in outcomes across time points (T0, T1, and T2) and determine if there is a statistically significant change over time. P-values < 0.05 were considered as statistically significant result.

## Results

### Descriptive statistics

#### Demographic or personal characteristics of pediatric oncology nurses

The study involved 39 pediatric ONs working at two nonprofit hospitals in the West Bank. Table 1A and 1B represent that the most of the nurses were women (84.6%, *n* = 33). The nurses ranged in age from 23.0 to 58.0, with an average age of about 31.7 years (SD = 8.56). The majority held a bachelor’s degree in nursing (79.5%, *n* = 31), while (15.4%, *n* = 6) had a master’s degree or higher, and (5.1%, *n* = 2) had a diploma. In terms of marital status, (64.1%, *n* = 25) were married, (33.3%, *n* = 13) were single, and one participant was divorced. Their overall nursing experience varied widely from 1.0 to 31.0 years (mean = 9.1, SD = 8.02), while their specific experience in pediatric oncology ranged from 1.0 to 21.0 years (mean = 6.8, SD = 5.30). Most of the nurses (92.3%, *n* = 36) worked as staff nurses; the rest held roles as coordinators (5.1%, *n* = 2) or administrators (2.6%, *n* = 1).

**Table 1A:** Demographic or personal characteristics of pediatric oncology nurses (*N* = 39)

Variable	M	SD	Min.	Max.
Age (Years)	31.7	8.56	23.0	58.0
Years of Experience as a Nurse	9.1	8.02	1.0	31.0
Years of Experience as a Pediatric Oncology Nurse	6.8	5.30	1.0	21.0

**Table 1B:** Demographic or personal characteristics of pediatric oncology nurses (*N* = 39)

Variable		*n*	%
Gender	Female	33	84.6
	Male	6	15.4
Educational Level	Diploma	2	5.1
	Bachelor Degree	31	79.5
	Master’s Degree or Higher	6	15.4
Marital Status	Single never married	13	33.3
	Married	25	64.1
	Divorced	1	2.6
What is Your Primary Position	Staff Nurse	36	92.3
	Nurse Coordinator	2	5.1
	Nurse Administrator	1	2.6

Of the nurses, 66.7% (*n* = 26) reported high satisfaction being a pediatric ONs, While 25.6% (*n* = 10) reported themselves as “neutral satisfied.” Regarding professional development, 66.7% (*n* = 26) of the participants had attended workshops or continuing education programs in pediatric oncology. However, only (5.1%, *n* = 2) had received formal training specifically in communication skills for working with pediatric oncology patients. Despite this, many nurses reported feeling capable in their communication skills: 64.1% (*n* = 25) indicated they were confident and 12.8% (*n* = 5) described themselves as very confident when interacting with parents. Another 23.1% (*n* = 9) expressed a neutral stance. All participants acknowledged that communication plays an important role in parental satisfaction with nursing care. Specifically, 53.8% (*n* = 21) believed it had a significant impact, 41.0% (*n* = 16) believed the impact was very significant, and only 5.1% (*n* = 2) perceived it as having a moderate effect (Table 2).

**Table 2:** Professional satisfaction, training, and communication impact of pediatric oncology nurses (*N* = 39)

Variable		*n*	%
How Satisfied are you with being a pediatric oncology nurse?	Dissatisfied	1	2.6
	Neutral	2	5.1
	Satisfied	26	66.7
	Very Satisfied	10	25.6
Have Ever attended Workshops or CEP related to pediatric oncology nursing	No	13	33.3
	Yes	26	66.7
Did you received a Formal Training or Education regarding communication skills for pediatric oncology Patient	No	37	94.9
	Yes	2	5.1
How confident are you in your current communication Skills when interacting with parents of pediatric oncology patients	Neutral	9	23.1
	Confident	25	64.1
	Very Confident	5	12.8
To what extent do you believe effective communication skills impact parents satisfaction with nursing care	Moderately	2	5.1
	Significantly	21	53.8
	Very Significantly	16	41.0

CEP: Continuous Education Program

### Nursing communication skills improvement

At baseline (T0), prior to the implementation of the intervention, nurses’ mean communication skills score was 3.84 (SD = 0.46), reflecting a moderate level of competency. Immediately after the intervention (T1), the mean score increased markedly to 4.53 (SD = 0.31), indicating a clear improvement in communication skills following the TAGEET model training. Two months post-intervention (T2), the mean score rose slightly further to 4.62 (SD = 0.26), suggesting that the gains were not only sustained but continued to progress over time, as detailed in Table 3.

**Table 3:** Descriptive statistics of communication skills among pediatric oncology nurses (*N* = 39)

Variable	M	SD	Min	Max
Nurses’ communication skills- Baseline (T0)	3.84	0.46	2.93	4.80
Nurses’ communication skills – Immediately (T1)	4.53	0.31	3.67	5.00
Nurses’ communication skills – Post 2 months (T2)	4.62	0.26	4.00	5.00

### Testing effect of the TAGEET intervention on communication skills over time

The assumptions for conducting repeated measures ANOVA on pediatric ONs communication skills were assessed. The primary assumption is sphericity, which requires the variances of the differences among all time points to be equal. This was tested using Mauchly’s Test of Sphericity.The results indicated that the assumption of sphericity was violated (*p* <.001). Consequently, a Greenhouse–Geisser correction (ε = 0.564) was applied to adjust for the violation and to reduce the risk of Type I error. The repeated measures ANOVA demonstrated a statistically significant effect of time on communication skills, F (1.13, 42.90) = 136.66, *p* <.001, partial η² = 0.782, indicating that approximately 78.2% of the variance in communication skills was attributable to changes over time (Table 4). The observed power was 1.000, confirming that the analysis had sufficient sensitivity to detect the effect.

**Table 4:** Communication skills tests within-subjective effects

Source		Type III Sum of Squares	df	Mean Square	F	Sig.	Partial Eta Squared	Observed Power^a^
Time	Sphericity Assumed	14.267	2	7.133	136.660	< 0.001	0.782	1.000
	Greenhouse-Geisser	14.267	1.129	12.637	136.660	< 0.001	0.782	1.000
	Huynh-Feldt	14.267	1.140	12.516	136.660	< 0.001	0.782	1.000
	Lower-bound	14.267	1.000	14.267	136.660	< 0.001	0.782	1.000

Effect size analyses further supported these findings. Cohen’s d was calculated by dividing the mean difference between two time points by the pooled standard deviation of the corresponding scores, thereby quantifying the magnitude of change in standardized units. The comparison between baseline (T0) and immediately post-intervention (T1) yielded a Cohen’s d of 1.78, reflecting a very large effect size and demonstrating that the training had an immediate and profound impact on nurses’ ability to communicate effectively. The contrast between baseline and two months post intervention (T2) was even greater, with Cohen’s d = 2.03, suggesting that the benefits of the intervention were not only sustained but also further consolidated over time. Finally, the comparison between T1 and T2 produced a Cohen’s d of 0.79, which, although smaller than the other contrasts, still represents a large effect, indicating continued refinement and strengthening of communication skills in the months following the training.

Bonferroni adjusted pairwise comparisons revealed statistically significant differences in communication skills scores between all-time points (Table 5). Scores at T1 were significantly higher than at T0 (mean difference = 0.692, *p* <.001), and scores at T2 were significantly higher than at T0 (mean difference = 0.781, *p* <.001), indicating substantial improvements following the intervention. Additionally, T2 scores were slightly but significantly higher than T1 (mean difference = 0.089, *p* <.001), suggesting a small but continued improvement between the post-intervention and follow-up measurements. The 95% confidence intervals for all comparisons did not cross zero, confirming the robustness of these differences.

**Table 5:** Bonferroni pairwise comparisons in communication skills scores between all time points

Time (I)	Time (J)	Mean Difference (I-J)	S.Error	Sig.^b^	95% Confidence Interval for Difference^b^	
					Lower Bound	Upper Bound
Baseline (T0)	T1	− 0.692^*^	0.062	< 0.001	− 0.848	− 0.536
	T2	− 0.781^*^	0.062	< 0.001	− 0.936	− 0.626
Immediately (T1)	T0	0.692^*^	0.062	< 0.001	0.536	0.848
	T2	− 0.089^*^	0.018	< 0.001	− 0.134	− 0.044
Post “2 months” (T2)	T0	0.781^*^	0.062	< 0.001	0.626	0.936
	T1	0.089^*^	0.018	< 0.001	0.044	0.134

*The mean difference is significant at the 0.05 level, ^b^Adjustment for multiple comparisons: Bonferroni

**Fig. 1:**
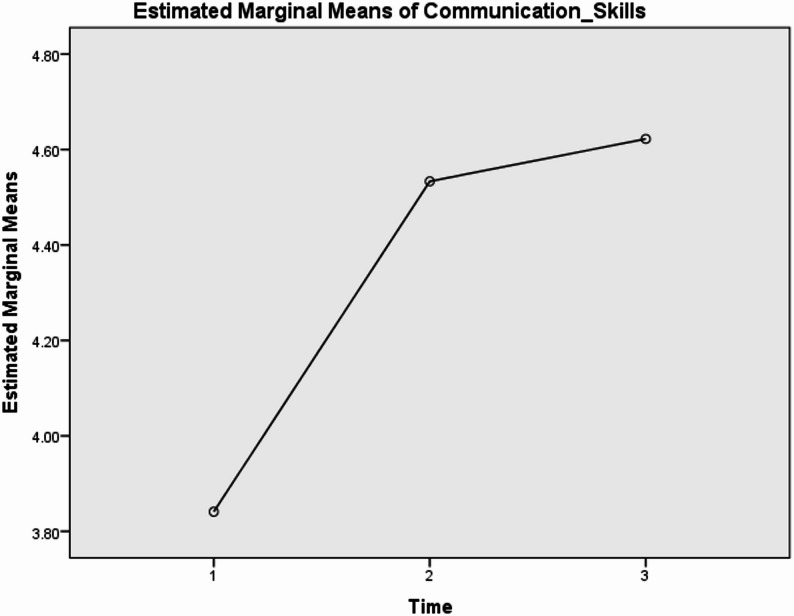
Profile plots of communication skills over three times (1: Baseline, 2: immediately, 3: post 2 months)

These statistical findings are visually supported by the estimated marginal means plot (Fig. 1), which shows a steep increase in communication skills from baseline (M ≈ 3.83) to immediately after intervention (M ≈ 4.54), followed by a smaller but positive increase to 2 months after intervention (M ≈ 4.63). The sharp initial gain reflects the immediate impact of the TAGEET model implementation, while the sustained upward trend suggests continued reinforcement of communication skills over time.

## Discussion

Pediatric ONs practice in a highly complex and emotionally demanding care environment, where effective therapeutic communication is fundamental to care quality [12, 13]. At baseline, nurses demonstrated a moderate level of communication competency, indicating reliance on experiential and intuitive approaches rather than structured, evidence-based frameworks. Relational behaviors such as respect, empathy, and attentiveness were the strongest domains, reflecting the centrality of caring values in nursing practice. These findings are consistent with literature identifying empathy and respect as core elements of therapeutic nurse and patient relationships and foundational to trust and collaboration [14].

However, notable gaps were observed in communication behaviors related to information exchange, shared decision-making, and future care planning. These deficits highlight challenges in actively engaging children and families as partners in care. Such patterns have been widely reported internationally, where nurses tend to excel in affective communication but demonstrate lower proficiency in structured engagement and decision facilitation [15, 16]. In pediatric oncology, communication is inherently complex, involving emotionally charged, triadic interactions among the child, parents, and HCPs, requiring both emotional sensitivity and cognitive clarity [17, 18]. These challenges are further reflected in Arab contexts, where communication practices are shaped by cultural norms, family roles, and protective attitudes toward children. For instance, evidence from Jordan indicates that families may prefer selective disclosure of information to protect the child, while emphasizing the importance of trust, compassion, and tailored communication in supporting coping and decision-making [19, 20]. In the Palestinian context, these challenges may be intensified by hierarchical clinical cultures, heavy workloads, time constraints, and the absence of standardized communication protocols, all of which limit opportunities for participatory dialogue with families.

Following implementation of the TAGEET communication model, nurses demonstrated significant improvements across all communication domains, with the greatest gains observed immediately after the intervention. The structured, stepwise design of the model appears to have provided clear and practical guidance that nurses could readily integrate into clinical interactions. Improvements were most pronounced in cognitive and interactive communication behaviors, including checking understanding, encouraging questions, involving parents in decisions, and using clear, accessible language. These findings suggest that TAGEET effectively addressed previously identified communication gaps and strengthened alignment with FCC principles.

Importantly, communication competency continued to improve two months after the intervention, indicating sustained behavioral change rather than short-term acquisition. This pattern suggests that the TAGEET model promoted internalization of communication skills through reflective practice and repeated application in real clinical setting [21]. Rather than reverting to intuitive habits, nurses appeared to consolidate and refine their skills over time, underscoring the model’s capacity to support long-term professional development.

The study findings align with global and regional evidence demonstrating the effectiveness of structured communication skills training in pediatric oncology and related settings [22–24]. Recent studies have further emphasized that structured communication training can significantly enhance clinicians’ ability to engage families, improve clarity of information exchange, and support shared decision-making processes [6, 12]. Notably, the greater improvements observed in cognitive and interactive domains, compared to relational domains, suggest that structured communication models primarily enhance technical communication skills rather than underlying caring attitudes. This distinction is clinically meaningful, as it indicates that even in settings where nurses demonstrate strong empathic orientation, targeted training can further strengthen communication effectiveness.

Beyond improvements in measured communication competency, the clinical significance of the TAGEET intervention lies in its potential to enhance patient and family-centered outcomes in pediatric oncology care. Effective communication has been consistently associated with improved parental understanding, greater trust in healthcare providers, enhanced emotional coping, and increased engagement in shared decision-making [6, 18]. In complex and emotionally demanding care trajectories, structured communication approaches may reduce uncertainty, improve satisfaction, and strengthen therapeutic relationships. The observed gains in cognitive and interactive communication domain, such as information clarity, checking understanding, and encouraging participation, represent key mechanisms through which communication interventions can influence patient experience and clinical quality. From a broader clinical perspective, strengthening nurses’ communication competencies may also contribute to improved treatment adherence, better care coordination, and fewer communication-related conflicts, all of which are critical in pediatric oncology settings [25, 26].

However, these findings should be interpreted with caution due to potential construct validity limitations. The reliance on self-reported measures, such as the CAT, may reflect increased awareness rather than consistent behavioral change in clinical practice. The absence of patient or parent reported outcomes further limits the ability to confirm whether improvements translated into meaningful enhancements in patient-centered care. In this study, collecting parent-reported data was not feasible due to practical considerations, including family vulnerability and respondent burden. Future research should therefore incorporate multi-method evaluation approaches, including observational assessments, parent-reported measures, and clinical quality indicators. Additionally, no significant association was found between marital status and communication competency, suggesting that professional training, clinical experience, and contextual factors rather than personal demographic characteristics more likely influence communication effectiveness.

This study has several limitations that should be acknowledged. The use of a single-arm pre–post design limits the ability to attribute observed improvements solely to the TAGEET intervention, as the absence of a control group does not fully exclude alternative explanation. Additionally, the sample size may have been insufficient to fully capture variability in communication competencies across different demographic characteristics, which may affect the generalizability of the findings. Nevertheless, the study provides valuable preliminary evidence from real-world pediatric oncology settings and highlights the feasibility and potential effectiveness of implementing a structured communication model in routine nursing practice.

## Conclusion

The implementation of the TAGEET communication model was associated with improved communication competency among pediatric oncology nurses, particularly in areas related to information exchange, engagement, and shared decision-making. These findings suggest that structured communication training can strengthen key aspects of therapeutic communication and support more effective nurse–family interactions in complex clinical settings.

While the results indicate the potential value of the TAGEET model in enhancing communication practice, they should be interpreted in light of the study’s design and reliance on self-reported measures. Further research incorporating patient- and parent-reported outcomes, observational assessments, and clinical indicators is needed to evaluate the extent to which these improvements translate into enhanced patient experience and quality of care.

Overall, integrating structured communication models such as TAGEET into routine nursing education and clinical practice may contribute to improving communication quality and supporting family-centered care in pediatric oncology settings.

## Data Availability

The datasets generated and/or analyzed during the current study are available from the corresponding author upon reasonable request and subject to ethical approval.

## Abbreviations

CAT: Communication assessment tool
ON: Oncology nurses
HCPs: Healthcare providers
JCI: Joint commission international
CEP: Continuing education programs
FCC: Family centered care
